# Association between labor and delivery pain and postpartum pain with symptoms and clinical diagnosis of postpartum depression in patients with overweight and obesity

**DOI:** 10.1007/s00404-022-06625-x

**Published:** 2022-06-04

**Authors:** Ezoza Rajabaliev, Kelsea LaSorda, Andrea Ibarra, Tanya Kenkre, Michele D. Levine, Grace Lim

**Affiliations:** 1 Department of Anesthesiology and Perioperative Medicine, University of Pittsburgh, Pittsburgh, PA, USA; 2 Department of Psychiatry, University of Pittsburgh, Western Psychiatric Institute and Clinic, Pittsburgh, PA, USA; 3 Department of Obstetrics, Gynecology, and Reproductive Sciences, University of Pittsburgh, Pittsburgh, PA, USA; 4 UPMC Magee Womens Hospital, 300 Halket Street Suite 3510, Pittsburgh, PA 15215, USA

**Keywords:** Pain, Depression, Postpartum, Diagnosis, Symptoms

## Abstract

**Purpose:**

Childbirth pain has been associated with the risk for postpartum depression. However, existing studies have been limited by the use of depression screening tools as outcomes, and none to date have used a structured clinical interview for DSM-V (SCID), which is considered the gold standard for psychiatric diagnoses. This study aimed to quantify the relationships between labor and postpartum pain and postpartum depression diagnosis detected by SCID, as well as depression symptoms detected by the Center for Epidemiological Studies Depression Scale (CESD) screening tool, among a high-risk cohort.

**Methods:**

The study was a secondary analysis of a prospective observational study of a cohort of women enriched for high risk for depression, i.e., pregnant women originally enrolled in a prospective study investigating factors leading to excessive gestational weight gain. Subjects were assessed prospectively for depression using both SCID and CESD at the third trimester and at 6 months postpartum. Overweight and obesity were defined as pre-gravid body mass index (BMI) ≥ 25 kg/m^2^. Both vaginal and cesarean deliveries were included in the cohort. Pain scores (0–10 numeric rating scale) during childbirth and after delivery were correlated with CESD and SCID. Propensity score matching was performed with propensity groups defined as those with low–moderate postpartum pain and those with high postpartum pain. The relationships between pain measures and 6-month postpartum depression diagnosis by SCID, and between pain measures and 6-month postpartum depression symptoms by CESD, were assessed by unweighted logistic regression and by logistic regression weighted by propensity score derived by average treatment effect (ATE) adjusted for baseline covariates.

**Results:**

There were 237 subjects in the cohort for analysis. Labor and postpartum pain were not associated with depression diagnosis by SCID at 6 months postpartum. However, postpartum pain, but not labor pain, was associated with depressive symptoms on the CESD at 6 months postpartum. Women with higher maximum postpartum pain scores had significantly higher odds of developing clinically significant postpartum depressive symptoms at 6 months, compared to those with lower pain scores in the unweighted model (OR: 1.3, 95% CI 1.0, 1.5; *P* = 0.005) and ATE-weighted models (OR: 1.2, 95% CI 1.0, 1.5; *P* = 0.03). Consistent with prior work, SCID and CESD were strongly associated, and 92.9% (13/14) of participants with postpartum depression diagnosis by 6-month SCID also showed high CESD symptomology, *P* < 0.0001).

**Conclusions:**

Although labor and postpartum pain were not associated with clinical diagnosis of depression (SCID) at 6 months postpartum, postpartum pain was linked to 6-month postpartum depression symptoms. Depressive symptoms are more likely to be exhibited in women with higher postpartum pain, potentially reflecting poorer birth recovery. The contribution of postpartum pain and depressive symptoms to overall patterns of poor recovery after childbirth should be assessed further.

## Introduction

Postpartum depression (PPD) is common, estimated to occur in one in five women after childbirth [1]. It can have both short- and long-term adverse effects on the mother, new-born, and community. All women after childbirth are at risk for developing PPD, but some known risk factors include physiological and psychosocial factors, hormonal changes, history of psychiatric disorders, or other emotional distress. Obesity is not only a known risk factor for depression, but obesity prior to pregnancy also increases the probability of developing postpartum depression [2]. Further, obesity is known to be associated with both depression and pain [3-5].

Questions have been raised about the effects of labor and postpartum pain and analgesia on maternal mental health outcomes such as depression. Neuraxial analgesia is the most effective form of pain management for labor, and some studies have found that epidural anesthesia reduces the risk for PPD [6, 7]. Other studies have found more conflicting results stemming from differences in pain management expectations and reality [8]. Further, obesity is known to be associated with both depression and pain [3-5]. Poorly managed pain represents an intervenable factor, so a true relationship between peripartum pain and perinatal depression would represent a potential complementary avenue for depression treatment interventions.

Clinically, assessments of depression routinely include clinical diagnosis as well as symptom self-report inventories; both assessments are valuable in the diagnosis and treatment of depression as well as symptom monitoring over time [5, 9]. A clinical diagnosis of depression is important because it is required to initiate medication therapies and clinical follow-up. Importantly, however, individuals with a history of depression may be at greater risk for experiencing depressive symptoms in the perinatal period [10]. Depressive symptoms are important aspects in health recovery after an acute event; for example, in perioperative pain and recovery outcomes, higher depressive symptoms have been associated with worse postoperative pain and recovery outcomes [11, 12]. To our knowledge, studies assessing the relationship between perinatal pain and depressive symptoms have not included the birthing person’s diathesis for depressive symptoms [13]. Longitudinal data on the experience of depression and postpartum pain are necessary to fully understand the unique contribution of pain to postpartum mood problems.

We included an enriched cohort of patients with overweight and obesity to quantify the relationship between labor and postpartum pain and PPD symptoms by CESD and diagnosis by Structured Clinical Interview for DSM-IV-TR Axis I Disorders (SCID). An enriched cohort of patients at high risk for depression, namely obese pregnant people who are at high susceptibility for perinatal depression, was included. Enriched cohort study designs have the advantage of increased prevalence of both risk-related factors and exposures, which enhance the power to identify such factors [14]. Our hypothesis was that women with higher labor and postpartum pain would have higher risk for clinical diagnosis of PPD by SCID as well as PPD symptoms by CESD.

## Methods

The study was approved by the University of Pittsburgh Institutional Review Board (IRB) (STUDY19040021). The requirement for written informed consent was waived by the IRB. The design was a secondary analysis of a previously conducted prospective observational trial during which written informed consent was obtained for trial participation. As previously reported [15, 16], the primary observational cohort study was designed to assess the relationship between loss of control over eating, and other psychosocial factors that may be related to gestational weight gain among individuals with obesity prior to pregnancy. Pregnant individuals with self-reported pre-gravid body mass index (BMI) greater than 25 were recruited at the end of the first trimester. Participants were excluded from the original cohort if they had type 1 diabetes, were currently participating in a weight management program, or if they were acutely suicidal requiring immediate treatment. Participants included in these secondary analyses were at least 14 years old, singleton pregnancy, and had pain scores recorded during labor, delivery, and postpartum hospital stay; individuals with fewer than 3 pain scores documented were excluded from analysis.

For the present study, an enriched cohort was used and consisted of patients at high risk for the depression outcome given that the population, pregnant people with BMI ≥ 25, was at high susceptibility for perinatal depression [14]. Briefly, the cohort was initially enrolled into a primary prospective cohort study called Relationship of Loss of Control Eating to Excessive Gestational Weight Gain (LEAP). LEAP enrolled pregnant patients and investigated factors leading to excessive gestational weight gain. Almost 50% of LEAP participants had a lifetime history of depression, and 7% of them had a diagnosis of depression at the time they were enrolled in the study. Because depression was more prevalent in this cohort, and because overweight and obesity—known risk factors for depression—was present in all participants, we chose this cohort for the present study and expected this sample to be able to detect associations between the predictors of interest (in this case, pain and depression).

In the primary study, after a brief phone screen to assess eligibility, eligible participants came to the clinic for an inperson baseline assessment, during which they completed questionnaires, height and weight measurements, and structured clinical interviews to assess lifetime psychiatric disorders. Participants were then followed longitudinally until 6 months postpartum. At the enrollment, at the end of the first trimester, and again at 6 months postpartum, depressive symptoms and diagnoses were assessed. For the present study, the electronic medical record was used to abstract the following data: age, estimated gestational age (weeks) at the time of delivery, gravidity, parity, BMI at delivery, mode of delivery, use of epidural labor analgesia. Manual record review was conducted to abstract co-existing medical conditions documented in labor and delivery records and coded in the diagnostic problem list by ICD-9. These were then grouped into the following categories: hypertensive disorders, anemia, history of miscarriage, chronic pain (encompassing cholestasis of pregnancy, pseudotumor cerebri, headache, irritable bowel syndrome, Reynaud’s, Sjogren’s disease, inflammatory pelvic disease, etc.), known fetal anomalies, and psychiatric disorders (including physical abuse, substance abuse, and insomnia, but excluding depression or anxiety). Data acquisition and verification were performed by two investigators (ER, KL) to ensure the reliability of the data. Any inconsistency or errors were either corrected or excluded from the study.

### Pain management regimens

Women with epidural labor analgesia were cared for at our tertiary hospital which employs standardized protocols for labor analgesia. Women with epidural labor analgesia receive a standardized medication regimen of bupivacaine 0.083% with fentanyl 2 mcg/mL and epidural fentanyl 100 mcg in divided doses. Initiation of analgesia included 3 mL of lidocaine 1.5% with 1:200,000 epinephrine as a test dose. The typical postpartum care for vaginal deliveries in this institution includes standardized acetaminophen and ibuprofen orally as needed, with oxycodone 5 or 10 mg as needed for severe breakthrough pain. The typical postpartum care for cesarean deliveries is scheduled acetaminophen and ibuprofen around the clock, as well as oxycodone 5–10 mg as needed for severe breakthrough pain.

### Pain variable calculations

Pain scores were abstracted from the medical record and partitioned to labor phase and postpartum phase (days 0–2 after delivery). During labor and in the postpartum units, patients have pain scores reported and documented throughout the hospital stay and are asked to rate their pain on a 0–10 numeric rating scale (NRS), where 0 is no pain and 10 is the worst pain imaginable. During labor, scores are recorded no fewer than once every 4 h. In the postpartum recovery units, scores are recorded no fewer than once every 8 h. For both labor and postpartum days 1–2, minimum and maximum pain scores for each subject were identified. Labor and postpartum pain burden was calculated by area under the curve (AUC), and percent improvement in pain (PIP), time-weighted (tw) PIP and tw NRS pain were calculated per our previously published methods [7, 17]. Time-weighted calculations enable assessments of pain burden independent of frequency of pain scores documented [7]. Briefly, AUC was calculated by the trapezoidal rule, while PIP and tw PIP were computed using the following equations:

$$\text{PIP}=\left(\frac{\text{baseline pain score}-\text{average intrapartum pain score}}{\text{baseline pain score}}\right)\times 100,$$

$$\text{tw NRS pain}=\frac{\sum \left({\text{NRS}}_{t-t_{0}}\times T_{i-i_{0}}\right)}{T_{i}},$$

$$\text{tw PIP}=\frac{\text{baseline pain score}-\text{TW pain}}{\text{baseline pain score}}$$

Baseline numeric pain score was defined as the level of pain rated just before the start of neuraxial anesthesia. Tw NRS pain scores estimated by dividing the sum of the product of the mean NRS and the time periods NRS were recorded by the sampling interval and were calculated to address any inconsistent sampling intervals for pain scores. Tw PIP pain scores were defined as the difference between the time-weighted pain score and baseline pain, divided by the baseline pain. Subjects with insufficient pain score data (fewer than 3 ratings) were excluded from the analysis. Multiple types of pain measures—encompassing pain burden and treatment response—were used to assess multiple dimensions of labor and postpartum pain to richly phenotype and evaluate potential pain factors and to generate hypotheses for future research directions.

### Depression variables

Lifetime psychiatric disorders were documented at the baseline interview (prior to 20 weeks gestation) by trained clinicians (master’s level or higher) using the Structured Clinical Interview for DSM-IV-TR Axis I Disorders: Non-Patient Version SCID-I/NP [18] adapted for DSM-5 diagnostic criteria. SCID diagnoses were used to create a binary outcome that captured depression diagnosis as present or absent. Concordance between diagnoses assessed via the SCID and other diagnostic tools is greater than 0.9, indicating excellent concordance [19]. In addition, patients were again interviewed at 6 months postpartum to capture the DSM disorders at 6 months postpartum.

Participants also reported depressive symptoms on the Center for Epidemiological Studies-Depression Scale (CESD) [20] at 6 months postpartum. CESD has a specificity of 90% and sensitivity of 86%, and its positive predictive value is 80% [21]. A positive screen was defined as a score greater than 16 on a scale of 0–60 [22].

### Statistical analysis

The primary outcome, SCID-Depression at 6 months (PPD at 6 months), was a composite measure of clinically diagnosed depression spectra, specifically major depressive disorder in the past month, current dysthymic disorder, depressive disorder not otherwise specified in the past month, and current depressive disorder. We estimated propensity scorederived average treatment effects (ATE) for patients classified as having high postpartum pain vs. low–moderate postpartum pain. Generalized boosting method propensity scores were calculated with measures of age, gravidity, parity, baseline BMI, anemia, chronic pain, diabetes, hypertension, known fetal anomalies, history of miscarriage, diagnosis of other psychiatric disorders, lifetime SCID assessment, labor analgesia, occurrence of vaginal delivery, and maximum labor pain score. Previous work has demonstrated these factors are related to both postpartum pain and postpartum depression, and we used them to calculate propensity scores in an effort to achieve balance with regard to these factors between the two postpartum pain groups (high vs. low–moderate) [7].

We compared the distribution of these measures among women with PPD at 6 months vs. those without PPD at 6 months with Fisher’s exact test (dichotomous measures) and Wilcoxon tests (continuous measures). We assessed the association between 6-month SCID and 6-month CESD with Fisher’s exact test. In unadjusted logistic regression models, we assessed the effect of chronic pain, psychiatric diagnosis, lifetime SCID assessment, SCID in the past month, labor analgesia, occurrence of vaginal delivery, minimum labor pain, maximum labor pain, AUC of labor pain, PIP labor pain, and twPIP labor pain each on four outcomes: unweighted 6-month SCID; ATE-weighted SCID; unweighted 6-month CESD; and ATE-weighted 6-month CESD.

## Results

A total of 237 women were included in the final analysis. The cohort of patients in our analysis included only individuals with BMI ≥ 25. Demographic and obstetric characteristics are shown in Table 1. At the time of enrollment, 32% (*n* = 75) of women had a level of depressive symptoms above the screen threshold according to CESD screening tool, and 7.6% (*n* = 18) met criteria for depression according to the SCID interview. Of 237 subjects, 5.9% (14) met criteria for depression with a perinatal onset according to SCID criteria and 16.5% (39) by CESD-positive screen at 6-month postpartum (Table 1). Results of the primary analysis showed that lifetime depression diagnosis by clinical interview was not associated with labor pain. However, women with more postpartum pain were more likely to report more depressive symptoms at 6 months postpartum (Table 2). That is, regardless of a person’s prior history of depression, postpartum pain experience was independently associated with increased depressive symptoms. Unadjusted logistic regression showed that neither labor nor postpartum pain predicted 6 months PPD diagnosis by SCID. Propensity score results showed no relationship between labor pain variables and 6-month PPD diagnosis by SCID [maximum labor pain unadjusted OR = 1.3 (95% CI 0.9, 1.9; *P* = 0.09); weighted by ATE OR = 1.3 (95% CI 0.9, 2.1; *P* = 0.18), maximum postpartum pain unadjusted OR = 1.3 (95% CI 0.9, 1.7; *P* = 0.10); weighted by ATE OR = 1.2 (95% CI 0.9, 1.6; *P* = 0.20)].

**Fig. 1:**
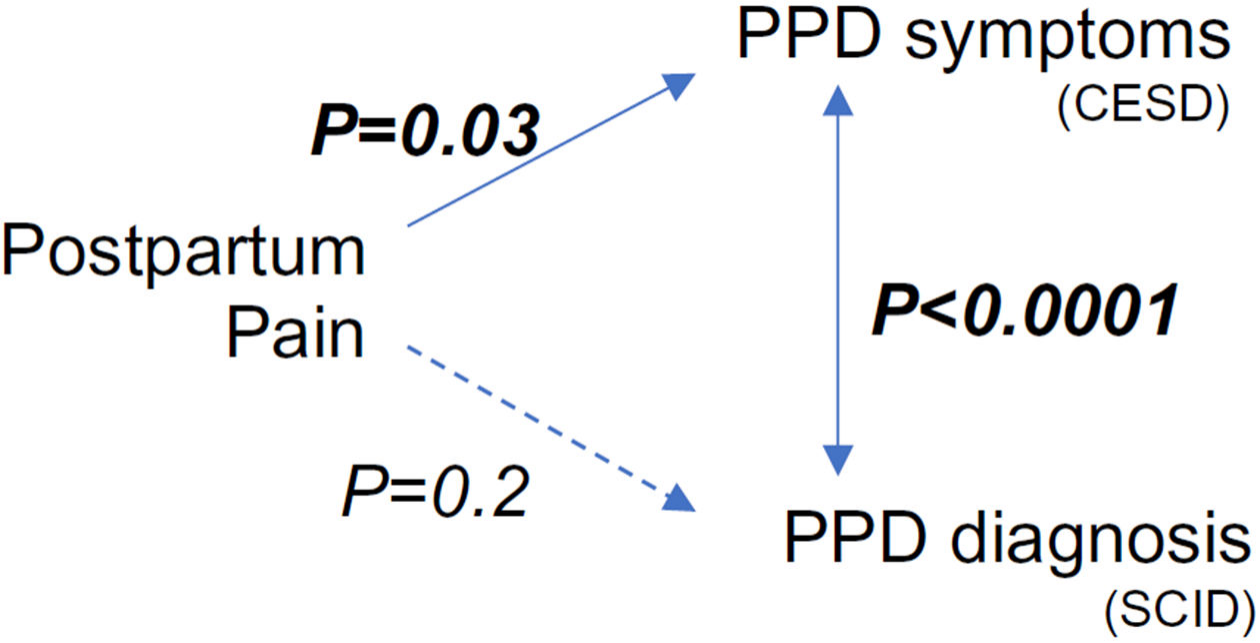
Summary of findings. Postpartum pain was associated with PPD symptoms, but not with PPD diagnosis. PPD symptoms and diagnosis were correlated. *PPD* postpartum depression, *CESD* Center for epidemiologic studies depression scale, *SCID* structured clinical interview for DSM-V

Higher postpartum pain significantly increased the odds of 6-month PPD symptoms by CESD; the maximum postpartum pain odds ratio was 1.3 (95% CI 1.0, 1.5; *P* = 0.005) in the unadjusted model and 1.2 (95% CI 1.0, 1.5; *P* = 0.03) in the ATE-weighted model (Table 3). Consistent with the CESD validation studies, there was a significant association between PPD by 6 months SCID and CESD: 92.9% of women with PPD by 6-month postpartum SCID also presented with high CESD symptomology, whereas 17.5% with undiagnosed PPD showed the symptoms of PPD (*P* < 0.0001) (Fig. 1).

## Discussion

The primary finding of this study suggests that although perinatal pain was not associated with clinical diagnosis of depression (SCID) at 6 months postpartum, postpartum pain was linked to 6-month postpartum depression symptoms measured by CESD. These results are notable especially considering the strong association between CESD and SCID. A clinical diagnosis of depression may not be influenced by labor or postpartum pain, but depressive symptoms are more likely to be exhibited in women with higher postpartum pain, perhaps reflecting poorer birth recovery. In support of this idea that poor pain control leads to poorer birth recovery, one study found that postpartum women with CESD scores > 16 (positive depression screen) were associated with reduced mother–infant bonding [23]. Poor elements of childbirth recovery—including pain and depressive symptoms—are important elements on which to focus future research. These findings do not preclude a potentially true relationship between peripartum pain and depression diagnosis.

Our study replicates the association between postpartum pain and the postpartum depression symptoms experienced by the cohort, as other studies have suggested. Eisenach et al. showed that women who experienced stronger acute postpartum pain within 36 h had a trifold risk of developing depression at 8 weeks postpartum, relative to those with less severe pain [11]. In a national analysis, Canadian Maternity Experiences Survey data revealed that participants who had unsettling perinatal pain in the first 3 months postpartum had 1.7 times greater odds (95% CI 1.2–2.5) of screening positive for depressive symptoms, compared to women with less severe perinatal pain [24]. Although our analysis found no link between clinical diagnosis of depression and peripartum pain, to confirm that women with worse perinatal pain experience depressive symptoms is clinically significant because these links between pain and negative affect or depression symptoms contribute to overall patterns of poor recovery after childbirth. Depressive symptoms should not be ignored just because they are not indicative of a clinical diagnosis. As a strong association was observed between CESD and SCID, we believe these findings suggest that depressive symptomatology is as important as a clinical diagnosis of depression, particularly in assessing the quality of childbirth recovery. Indeed, several postoperative and postpartum quality of recovery instruments include raw or global assessments of mood or emotional states, such as feeling anxious or depressed, to assess the emotional dimensions of recovery and quality of life. [25-27]

Prior published research suggests an interaction between the actual use and intention to use of epidural analgesia and PPD at 6 weeks, as reported by Orbach-Zinger who found a significant negative additive relationship between unmatched expectations when women delivered with labor epidural anesthesia (LEA) when they had not planned to do so, or vice versa [8]. However, in multivariable logistic regression analysis, the relationship between intended and actual use of LEA lowered the adjusted odds of PPD. The authors suggested that the factors such as negative physiological aspects of delivery, unpleasant emotions, or unparalleled expectations had an impact on the interaction between unplanned LEA and PPD. Our findings seem to support these ideas as well: our observed association between pain and PPD symptoms but notdepression diagnosis, suggest that there may be negative emotions or symptoms after delivery that are associated with worsepain and recovery, but not necessarily a clinical diagnosis of depression. Another study hypothesized that there was a strong correlation between different types of low back pain (lumbar pain, posterior pelvic pain, and combined pain) and perinatal depressive symptoms [28]. It was concluded that the prevalence of prenatal, perinatal, and postnatal depressive symptoms, assessed by Edinburgh postnatal depression scale, varied depending on the type of low back pain women experienced. These studies are overall in line with the results of our study and highlight the negative impact of the pain experience of women during pregnancy and birth on depressive symptoms.

The interpretation of these results should be in the context of the limitations of this study. The rates of depression were lower than quoted in the general population and could have limited the ability for our analysis to detect a true relationship with clinical depression diagnosis. As a secondary analysis, the intent of the primary study was not to draw links between pain and depression. Further, reliance on data abstracted from medical record has limitations inherent in medical record analyses, such as missing clinical labor and postpartum pain data placing limitations on robust analyses; however, our time-weighted analyses are able to account for variations in sampling frequencies. Notable items that were not available in the original data set, and which could not be included in this analysis, included duration of labor, induction of labor, instrumental delivery, mode of delivery, emergency deliveries, postoperative complications, and additional diagnoses within our cohort such as anxiety and trauma or post-traumatic stress disorder. These omissions could potentially influence the limited ability to detect a potential true relationship between more severe pain and psychological outcomes. Antepartum pain experiences could not be accounted for in this analysis. It is notable that the number of positive diagnoses of depression at 6 months postpartum in this cohort was only 5%, only 14 cases out of 257. We were surprised to find that this was below average rates in the general population and may potentially reflect a sampling bias. It is possible that the final cohort may reflect a sample that had less severe depressive disease and were more likely to continue with study procedures. However, we judge that despite the potential for sampling bias there remains value in these study results, because it is the first to our knowledge to address the issue of depression diagnosis versus symptoms in relationships with labor and postpartum pain. We judge that depression symptoms are as important as diagnoses in assessing depression outcomes, and symptom measurement is valuable because perinatal depression is also managed based on symptoms. Altogether, our findings support the need for additional research in a larger cohort on the interactions between poorly controlled pain, depression symptoms—not necessarily a diagnosis only—and quality of childbirth recovery in new mothers.

In this exploratory analysis, we used a cohort of patients, which included only individuals with overweight/obesity, thus being at an especially high risk for depression. Our findings suggest that postpartum pain is associated with postpartum depressive symptoms, but not a clinical diagnosis of depression. Findings of this study need replication and do not exclude the potential for a true relationship between postpartum pain and clinical diagnosis of depression. Future investigations can focus on assessing the relationships between postpartum pain, depressive symptoms and diagnoses, and overall quality of recovery of women after childbirth, especially among high-risk patients such as those with obesity. The relative contribution of postpartum pain and depressive symptoms to overall patterns of recovery after childbirth requires additional characterization.

## Figures and Tables

**Table 1:** Demographic and obstetric characteristics

Variable	PPD at 6 months postpartum [*N* = 48]	No PPD at 6 months postpartum [*N* = 189]
Age	27.26 ± 5.09	28.48 ± 5.35
BMI at delivery (kg/m^2^)	38.60 ± 6.98	37.55 ± 6.63
Estimated gestational age at delivery (weeks)	38.93 ± 3.04	38.49 ± 2.93
Gravidity	3.65 ± 2.61	2.85 ± 2.09
Parity	1.42 ± 1.14	1.15 ± 1.40
Vaginal delivery	64.29% [27]	68.57% [120]
Unscheduled cesarean delivery	33.33% [16]	16.40% [31]
Epidural labor analgesia utilization	65.85% [27]	70.93% [122]
Chronic pain	35.71% [15]	39.31% [68]
Anemia	19.05% [8]	17.92% [31]
History of miscarriage	33.33% [14]	26.63% [45]
Known fetal anomalies	2.33% [1]	3.51% [6]
Hypertension	2.79% [6]	23.12% [40]
Psychiatric diagnoses (excluding depression or anxiety)	69.77% [30]	50.29% [86]
SCID and CESD measurements at enrollment time and 6 months postpartum		
Variable	At time of enrollment	At 6-months postpartum
CESD	75/237 (32%)	39/223 (17.5%)
SCID	18/237 (8%)	14/237 (5.9%)

Data are presented as means ± standard deviation or frequency and percentages

*PPD* Postpartum depression, *BMI* Body mass index, *CESD* Center for epidemiologic studies depression scale (screen), *SCID* structured clinical interview for DSM-V (diagnosis)

**Table 2:** Unadjusted logistic regression models predicting SCID and CESD at 6 months postpartum

	Outcome			
	SCID-unweighted (6 months)	SCID ATE weighted (6 months)	CESD-unweighted (6 months)	CESD ATE-weighted (6 months)
Predictor				
Chronic pain	OR = 0.18	OR = 0.22	OR = 1.01	OR = 1.01
	95% CI: 0.02, 1.40	95% CI: 0.03, 1.74	95% CI: 0.50, 2.05	95% CI: 0.49, 2.11
	*p* value = 0.10	*p* value = 0.15	*p* value = 0.98	*p* value = 0.98
Psychiatric diagnosis	OR = 1.62	OR = 1.53	***OR = 4.01***	***OR = 3.61***
	95% CI: 0.53, 5.02	95% CI: 0.48, 4.92	***95% CI: 1.92, 8.41***	***95% CI: 1.68, 7.76***
	*p* value = 0.40	*p* value = 0.48	***p value = 0.0002***	***p value = 0.001***
SCID0- lifetime	OR = 2.82	OR = 2.79	***OR = 4.28***	***OR = 4.17***
	95% CI: 0.91, 8.71	95% CI: 0.87, 8.99	***95% CI: 2.17, 8.44***	***95% CI: 2.05, 8.47***
	*p* value = 0.07	*p* value = 0.09	***p value < 0.0001***	***p value < 0.0001***
SCID0- past month	OR = 3.54	OR = 3.74	***OR = 18.45***	***OR = 17.31***
	95% CI: 0.69, 18.22	95% CI: 0.69, 20.25	***95%CI: 3.84, 88.74***	***95%CI: 3.52, 85.21***
	*p* value = 0.13	*p* value = 0.13	***p value = 0.0003***	***p value = 0.001***
Labor analgesia	OR = 1.31	OR = 1.20	OR = 0.91	OR = 0.84
	95% CI: 0.59, 2.90	95% CI: 0.61, 2.38	95% CI: 0.54, 1.51	95% CI: 0.49, 1.45
	*p* value = 0.51	*p* value = 0.60	*p* value = 0.70	*p* value = 0.53
Vaginal delivery	OR = 0.63	OR = 0.84	OR = 0.89	OR = 1.12
	95% CI: 0.21, 1.90	95% CI: 0.27, 2.59	95% CI: 0.45, 1.75	95% CI: 0.56, 2.24
	*p* value = 0.41	*p* value = 0.75	*p* value = 0.74	*p* value = 0.76
Labor pain-min	OR = 1.04	OR = 1.02	OR = 0.96	OR = 0.96
	95% CI: 0.85, 1.27	95% CI: 0.84, 1.22	95% CI: 0.85, 1.08	95% CI: 0.85, 1.08
	*p* value = 0.72	*p* value = 0.86	*p* value = 0.47	*p* value = 0.46
Labor pain-max	OR = 1.34	OR = 1.34	OR = 1.14	OR = 1.13
	95% CI: 0.95, 1.88	95% CI: 0.88, 2.05	95% CI: 0.99, 1.32	95% CI: 0.98, 1.31
	*p* value = 0.09	*p* value = 0.18	*p* value = 0.08	*p* value = 0.10
Labor pain-AUC	OR = 1.00	OR = 1.00	OR = 1.00	OR = 1.00
	95% CI: 1.00, 1.00	95% CI: 1.00, 1.00	95% CI: 1.00, 1.00	95% CI: 1.00, 1.00
	*p* value = 0.86	*p* value = 0.76	*p* value = 0.35	*p* value = 0.10
Labor pain-PIP	OR = 1.00	OR = 1.00	OR = 1.00	OR = 1.00
	95% CI: 1.00, 1.01	95% CI: 1.00, 1.01	95% CI: 1.00, 1.00	95% CI: 1.00, 1.00
	*p* value = 0.49	*p* value = 0.35	*p* value = 0.50	*p* value = 0.62
Labor pain-PIPTW	OR = 1.01	OR = 1.01	OR = 1.00	OR = 1.00
	95% CI: 0.98, 1.04	95% CI: 0.98, 1.05	95% CI: 0.98, 1.01	95% CI: 0.98, 1.01
	*p* value = 0.39	*p* value = 0.43	*p* value = 0.48	*p* value = 0.57
Postpartum pain-min	OR = 1.28	OR = 1.23	OR = 1.20	OR = 1.16
	95% CI: 0.92, 1.78	95% CI: 0.91, 1.67	95% CI: 0.96, 1.51	95% CI: 0.93, 1.46
	*p* value = 0.15	*p* value = 0.17	*p* value = 0.11	*p* value = 0.19
Postpartum pain-max	OR = 1.28	OR = 1.19	***OR = 1.27***	***OR = 1.22***
	95% CI: 0.95, 1.71	95% CI: 0.91, 1.55	***95% CI: 1.08, 1.50***	***95% CI: 1.02, 1.45***
	*p* value = 0.10	*p* value = 0.20	***p value = 0.005***	***p value = 0.03***
Postpartum pain-AUC	OR = 1.00	OR = 1.00	**OR = 1.00**	**OR = 1.00**
	95% CI: 1.00, 1.00	95% CI: 1.00, 1.00	**95% CI: 1.00, 1.00**	**95% CI: 1.00, 1.00**
	*p* value = 0.11	*p* value = 0.15	**p value = 0.01**	**p value = 0.04**
Postpartum pain-PIP	OR = 1.00	OR = 1.00	**OR = 1.00**	**OR = 1.00**
	95% CI: 1.00, 1.01	95% CI: 1.00, 1.01	**95% CI: 1.00, 1.00**	**95% CI: 1.00, 1.00**
	*p* value = 0.53	*p* value = 0.19	**p value = 0.03**	**p value = 0.05**
Postpartum pain-PIPTW	OR = 1.00	OR = 1.00	OR = 1.00	OR = 1.00
	95% CI: 1.00, 1.01	95% CI: 1.00, 1.01	95% CI: 1.00, 1.00	95% CI: 1.00, 1.00
	*p* value = 0.51	*p* value = 0.18	*p* value = 0.15	*p* value = 0.24

Bolded values reflect P < 0.05

*ATE* Average treatment effect from propensity score analysis, *OR* odds ratio, *CI* Confidence interval, *CESD* Center for epidemiologic studies depression scale (screen), *SCID* Structured clinical interview for DSM-V (diagnosis), *AUC* Area under the curve, *PIP* Percent improvement in pain, *PIPTW* Percent improvement in pain time-weighted calculation

**Table 3:** Association between SCID diagnosis and CESD screen at 6 months postpartum

	No SCID diagnosis	SCID diagnosis	*P* value
	*N* = 223	*N* = 14	
CESD positive	39/223 (17.5%)	13/14 (92.9%)	**< 0.0001**

Bolded values reflect P < 0.05

Data are presented as rates (percentages)

*CESD* Center for epidemiologic studies depression scale (screen). A positive screen is a score ≥ 16. *SCID* Structured clinical interview for DSM-V (diagnosis)
